# Lipid and metabolite correlation networks specific to clinical and biochemical covariate show differences associated with sexual dimorphism in a cohort of nonagenarians

**DOI:** 10.1007/s11357-021-00404-3

**Published:** 2021-07-29

**Authors:** Francesca Di Cesare, Leonardo Tenori, Gaia Meoni, Anna Maria Gori, Rossella Marcucci, Betti Giusti, Raffaele Molino-Lova, Claudio Macchi, Silvia Pancani, Claudio Luchinat, Edoardo Saccenti

**Affiliations:** 1 Magnetic Resonance Center (CERM), University of Florence, Sesto Fiorentino, Italy; 2 Department of Chemistry “Ugo Schiff”, University of Florence, Sesto Fiorentino, Italy; 3 Giotto Biotech srl, Florence, Italy; 4 Department of Experimental and Clinical Medicine, University of Florence, Florence, Italy; 5 Atherothrombotic Unit, Careggi University Hospital, Florence, Italy; 6 IRCCS Fondazione Don Carlo Gnocchi, Florence, Italy; 7 Consorzio Interuniversitario Risonanze Magnetiche di Metallo Proteine (CIRMMP), Sesto Fiorentino, Italy; 8 Laboratory of Systems and Synthetic Biology, Wageningen University & Research, Wageningen, the Netherlands

**Keywords:** Aging, Differential network analysis, Lipidomics, Metabolomics, Network inference, Nuclear magnetic resonance, Sexual dimorphism

## Abstract

This study defines and estimates the metabolite-lipidic component association networks constructed from an array of 20 metabolites and 114 lipids identified and quantified via NMR spectroscopy in the serum of a cohort of 355 Italian nonagenarians and ultra-nonagenarian. Metabolite-lipid association networks were built for men and women and related to an array of 101 clinical and biochemical parameters, including the presence of diseases, bio-humoral parameters, familiarity diseases, drugs treatments, and risk factors. Different connectivity patterns were observed in lipids, branched chains amino acids, alanine, and ketone bodies, suggesting their association with the sex-related and sex-clinical condition-related intrinsic metabolic changes. Furthermore, our results demonstrate, using a holistic system biology approach, that the characterization of metabolic structures and their dynamic inter-connections is a promising tool to shed light on the dimorphic pathophysiological mechanisms of aging at the molecular level.

**Supplementary Information:**

The online version contains supplementary material available at 10.1007/s11357-021-00404-3.

## Introduction

Nonagenarian and centenarian people represent a considerable increasing fraction of the world population, concentrated, above all, in economically developed countries [1, 2]. *In Europe, Italy and France hold the record for the number of living centenarians. According to a 2019 statistic, in Italy, 1% of the population is 90 years older or more, and between 2000 and 2019 the number of centenarians (85% women) increased from 11000 to more than 14456, and the number of ultra-centenarians (>105 years, 94% women) increased 136%, from 4*72 to 1112 [3].

Aging is associated with irreversible variations in biological, pathophysiological, and psychological dynamics [4–8]: these age-related changes result in a decline of cognitive, motor, and sensory functions and in an increase of susceptibility to disease and disease frequency, a poor quality of life, and increased mortality [9, 10], defining a clinically and biologically heterogeneous population.

Many different fundamental biological processes, such as inflammation, cellular and immune senescence, mitochondrial dysfunction, and reduced resistance to oxidative stress are the main mechanisms at the basis of the aging process [10]. The progressive decline of physiological functions reflects changes happening at the molecular, organelle, cell, tissue, and, finally, the whole organism level [11, 12]. Although individually these biochemical and molecular alterations underlying these processes may have only a modest effect on aging, taken together they involve a complex network of biomolecular mechanisms acting across multiple organs and at different molecular levels [13]*.*

Accumulation of molecular damage has been proposed to be among the mechanisms driving aging [14–16], and this may include not only oxidative stress and DNA mutations, but also errors in protein synthesis and by-products of enzymatic reactions [17]. In this light, the use of *high-throughput omics techniques, like genomics, transcriptomics, proteomics, and metabolomics, offers a great promise for the* understanding of the mechanisms that underlie aging [11, 18, 19]. In particular, the analysis of metabolic signatures associated with age and the comprehensive characterizing and understanding of the structures, functions, and interactions between metabolites and lipidic components, can shed light on the potential mechanism that could influence aging and longevity.

Nuclear magnetic resonance (NMR)-based metabolomics offers the possibility to quantify and investigate hundreds of various metabolites, lipid fractions and sub-fractions [20–23], detectable in biofluids, providing a global image of the complex metabolic, biological and biophysical processes associated with health [10, 22, 24] and disease [23, 25–27].

Integrative analysis of NMR-based metabolomics data using systems biology approaches focusing on the interactions and relationships among biochemical molecules like protein and metabolites can offer a holistic representation of the metabolic structures, indispensable for the understanding of the molecular mechanism underlying aging [28, 29].

Networks and network analysis of blood metabolites, lipid fractions and sub-fractions association networks are fundamental tools to extract information on the status of a biological system since correlation among metabolites and lipids concentration profiles can be used to model and to infer, at least partially, the structure of the underlying biological network [30]. In addition, these network models can be linked to clinical information such as biochemical parameters, risk factors, comorbidities, allowing the analysis of the relationships existing between different network structures and patient clinical characteristics.

In this work, we take an integrative approach to investigate the associations between metabolite and lipoprotein/lipid networks and the range of clinical, biochemical, environmental, socio-demographic parameters collected on a cohort of 355 nonagenarians from the Italian Mugello study [31]. The goal of the present analysis is twofold: first, we wanted to understand the association between different networks’ structures and sex, because of its relevance for gender medicine of aging [32, 33]; second, we wanted to explore the complex web of relationship existing between clinical parameters, risk factors, and comorbidities and blood metabolites and lipids association patterns.

## Material and methods

### Study description

**Fig. 1:**
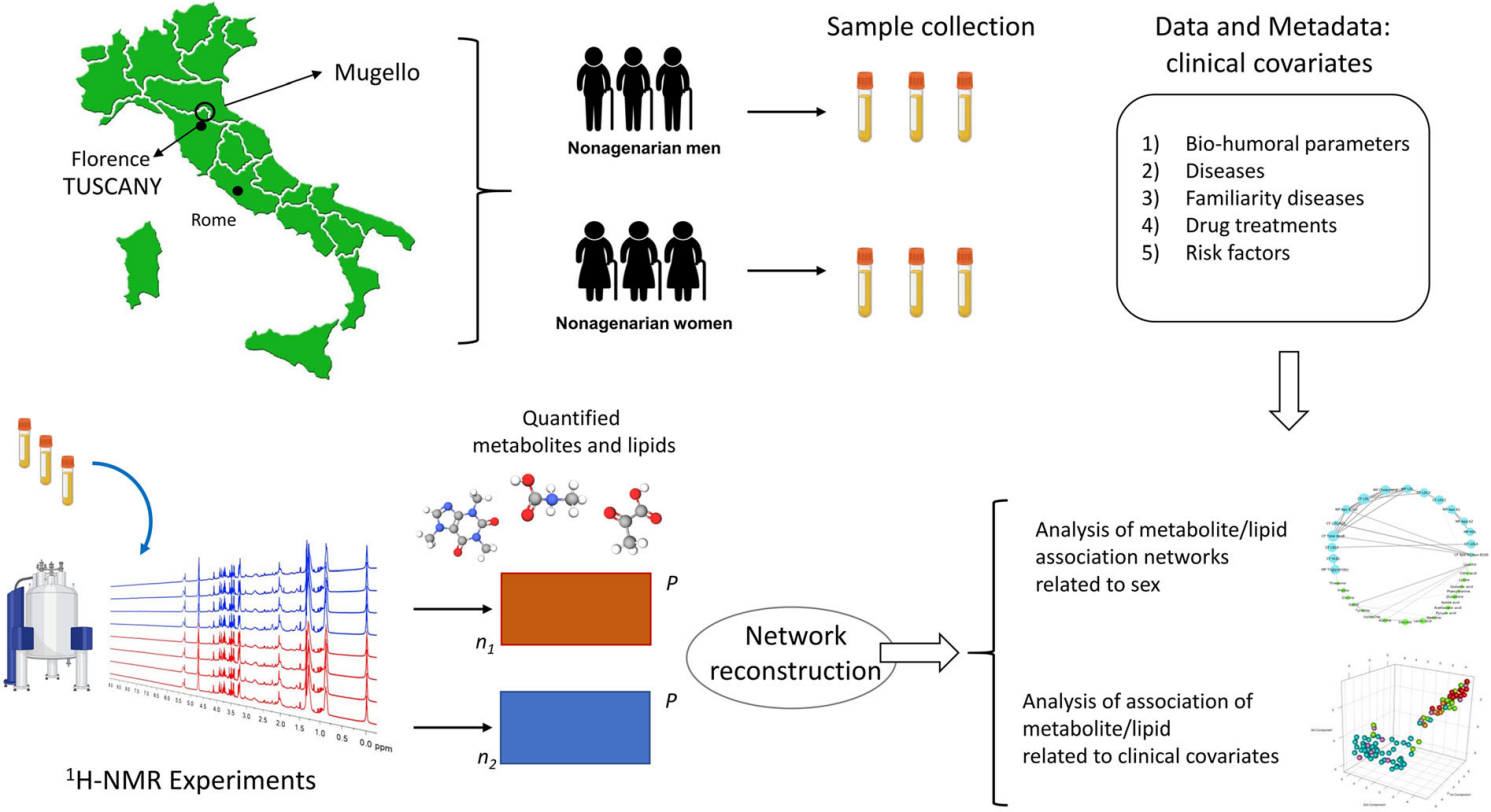
Graphical overview of the study and the data analysis strategy used in this study. Bio-humoral parameters refer to biochemical information such as blood count, mean cell volume, mean cell hemoglobin, thyroid hormones; see Table 1 for more details. Data are from the Mugello study [31]

Samples were collected from the participants in the Mugello study, an epidemiological survey conducted from January 2010 to December 2011 in the Mugello area (north-eastward of Florence, Tuscany, Italy, see Fig. 1) [31]. The original study comprised 356 subjects—of which 96 men (27%) and 260 women (73%), with an age range of 84–103 years and of 88–105 years and with mean age 92.6 (± 3.4) and 93.2 (± 3.2) years, respectively. All participants, at the time of the cross-sectional survey, were subjected, by a trained physician, to a series of home-based structured interviews and medical examinations regarding clinically relevant geriatric conditions. Blood samples were also collected to perform routine laboratory tests. We refer the reader to the original publication for more details on the study design and the protocols that have been followed [31].

### Overview of clinical variables

For the analysis presented in this article, we selected a sub-set of *M*=101 clinical covariates which were grouped into 5 categories:

1. Diseases (*m*=20, dichotomous variables: 0-1): describing the existence of a specific medical condition, *i.e.*, myocardial infarction, congestive heart failure, peripheral vascular disease, hemiplegia, hypertension, dyslipidemia, dementia, cerebrovascular disease, diabetes (with and without organ damage), cancer, leukemia, disability, elderly depression (evaluated using the 15-item Geriatric Depression Scale screening questionnaire validated for the geriatric population [34]), cognitive impairment (evaluated using the Mini-Mental State Examination validated questionnaire [35]) and motor impairment (evaluated using Short Physical Performance Battery and Time up and go questionnaires [36, 37]).
2. Familiarity diseases (*m*=5, dichotomous variables: 0-1): describing the existence of familiarity for cardiovascular, respiratory, and cerebrovascular diseases, dementia, and cancer.
3. Drugs treatments (*m*=13, dichotomous variables: 0-1): indicating the presence of ongoing pharmacological treatment for diseases described above.
4. Risk factors (*m*=12, continuous and dichotomous variables) including socio-demographic variables (age, education, sleep alertness, civil status, living with and smoke habit) and physical parameters (Body Mass Index [38–40], Windsor index or systolic ankle pressure measured using Ankle-brachial index [41, 42], Physical activity scale for elderly score [43], Handrig index [44], Mediterranean Diet Score [45, 46]).
5. Bio-humoral parameters (*m*=51: continuous and dichotomous) including, *i.e.*, complete blood count, mean cell volume, mean cell hemoglobin, thyroid hormones, cholesterol, HDL, LDL, and glycemia.

The variables considered and associated statistics are listed in Table 1.

**Table 1:** Descriptive statistics of clinical variables, divided into five separated categories, stratified by sex. For continuous variables the mean ± SD (standard deviation) is reported. *GDS* geriatric depression scale, *MMSE* Mini-Mental State Examination, *SPPB* short physical performance battery, *MCV* mean corpuscular volume, *MCH* mean corpuscular hemoglobin, *MCHC* mean corpuscular hemoglobin concentration, *RDWCV* red blood cells distribution width coefficient of variation, *RDWSD* red blood cells distribution width standard deviation, *PDW* platelet distribution width, *MPV* mean platelet volume, *PLCR* platelet large cell ratio, *GOT-AST* aspartate aminotransferase, *GPT-ALT* alanine transaminase, *γ-GT* gamma-glutamyl transferase, *TSH* thyroid-stimulating hormone, *WBCs* white blood cells, *RBCs* red blood cells, *HCT* hematocrit, *HbA1C* hemoglobin A1c, *CRP* C-reactive protein, *ACE* Angiotensin-converting enzyme, *ABI* Ankle-Brachial Index, *BMI* Body Mass Index, and *MDS* myelodysplastic syndromes

		women (*n* = 259, 73.0 %)	men (*n* = 96, 27.0 %)
**Diseases**	Myocardial infarction (%)	15.4	12.5
	Congestive heart failure (%)	23.2	15.6
	Peripheral vascular diseases (%)	22.4	11.5
	Hypertension (%)	55.9	58.3
	Dyslipidemia (%)	12.4	6.3
	Dementia (%)	13.5	13.5
	Diabetes (%)	12.7	15.6
	Diabetes without organ damage (%)	7.3	13.5
	Diabetes with organ damage (%)	5.8	2.1
	Cancer (%)	13.9	9.4
	Leukemia (%)	0.4	0.0
	Disability (%)	62.6	88.5
	Motor impairment code	9.0 ± 6.7	7.5 ± 7.4
	GDS code	0.6 ± 0.5	1.8 ± 0.8
	Depression (%)	58.7	77.1
	MMSE (%)	56.4	50.0
	SPPB (%)	63.3	64.6
	Time up and go (%)	58.7	64.6
	Hemiplegia (%)	0.8	1.0
	Cerebrovascular diseases (%)	21.8	20.8
**Bio-humoral parameters**	MCV (FL)	90.4 ± 5.3	90.3 ± 6.2
	MCH (pg.)	29.7 ± 2.9	29.7 ± 2. 6
	MCHC (g/dL)	33.0 ± 1.0	33.0 ± 1.1
	RDWCV (fL)	14.7 ± 1.3	14.8 ± 1.4
	RDWSD (fL)	47.3 ± 4.1	47.5 ± 5.8
	PDW (fL)	13.3 ± 2.2	13.9 ± 2.5
	MPV (fL)	10.6 ± 1.0	10.9 ± 1.0
	PLCR	29.6 ± 6.9	31.3 ± 6.5
	GOT-AST (IU/L)	20.2 ± 9.4	20.1 ± 5.9
	GPT-ALT (IU/L)	15.0 ± 9.4	14.2 ± 5.2
	γ-GT (IU/L)	25.2 ± 27.6	15.6 ± 4.1
	Neutrophil (x10^3^/μL)	4.0 ± 2.7	4.1 ± 1.7
	Lymphocyte (x10^3^/μL)	1.8 ± 0.9	1.8 ± 0.7
	Monocyte (×10^3^/μL)	0.5 ± 0.2	0.5 ± 0.2
	Eosinophil (x10^3^/μL)	0.2 ± 0.1	0.2 ± 0.1
	Basophil (x10^3^/μL)	0.02 ± 0.02	0.03 ± 0.03
	Neutrophyl – formula	59.7 ± 9.5	60.7 ± 9.7
	Lymphocyte – formula	28.9 ± 8.8	27.7 ± 8.5
	Monocyte – formula	7.7 ± 2.3	7.9 ± 2.4
	Eosinophil – formula	3.2 ± 2.1	3.4 ± 2.0
	Basophil – formula	0.4 ± 0.4	0.4 ± 0.3
	Creatinine (mg/dL)	1.0 ± 0.5	1.1 ± 0.7
	Neutrophyl/lymphocyte	0.5 ± 0.3	0.5 ± 0.3
	Platelets (x10^3^/μL)	219.6 ± 93.9	206.5 ± 64.0
	Na^+^ (mE/ql)	138.9 ± 3.0	138.6 ± 2.9
	K^+^ (mEql)	4.3 ± 0.5	4.4 ± 0.5
	Cl- (mEql)	101.7 ± 7.8	102.0 ± 4.0
	Total proteins (g/dL)	6.4 ± 0.6	6.5 ± 0.7
	Albumin (g/dL)	56.2 ± 4.6	56.1 ± 4.8
	α1-G (g/dL)	3.9 ± 1.3	4.0 ± 1.5
	α2-G (g/dL)	12.0 ± 2.0	12.0 ± 1.8
	β-G (g/dL)	12.2 ± 1.7	12.3 ± 1.9
	γ-G (g/dL)	15.6 ± 3.7	15.6 ± 4.1
	A/G	1.3 ± 0.3	1.3 ± 0.3
	T3 (pg/mL)	2.8 ± 0.5	2.9 ± 0.6
	T4 (ng/dL)	0.9 ± 0.2	0.9 ± 0.3
	TSH (μUI/mL)	2.3 ± 6.3	2.1 ± 4.0
	WBCs (x10^3^/(μL)	6.3 ± 1.9	6.7 ± 2.1
	RBCs (x10^6^/(μL)	4.3 ± 0.6	4.4 ± 0.6
	Hemoglobin (g/dL)	12.9 ± 1.5	12.9 ± 1.7
	HCT (%)	38.9 ± 4.8	39.0 ± 4.8
	glycemia (mg/dL)	94.6 ± 26.7	93.3 ± 22.7
	HbA1C (g/Hb)	5.6 ± 0.8	5.6 ± 0.8
	Total cholesterol (mg/dL)	190.5 ± 42.0	192.9 ± 41.2
	Cholesterol (mg/dL)	0.4 ± 0.5	0.4 ± 0.5
	HDL (mg/dL)	57.4 ± 16.4	59.0 ± 18.7
	LDL (mg/dL)	110.3 ± 33.7	111.0 ± 32.8
	Triglycerides (mg/dL)	113.9 ± 47.5	115.1 ± 55.1
	CRP (mg/L)	0.9 ± 2.1	1.2 ± 2.8
	CRP (%)	54.4	64.6
	Inflammatory protein (mg/L)	9.2 ± 20.6	11.9 ± 27.5
	Benzodiazepine (%)	15.4	18.8
**Drug treatment**	Antidepressant (%)	18.5	19.8
	Diuretics (%)	52.5	44.8
	Beta-blockers (%)	10.4	15.6
	Ca^++^ channel blockers (%)	19.7	16.7
	ACE inhibitors (%)	39.0	35.4
	Vasodilators nitrates (%)	24.3	21.9
	Oral anticoagulant (%)	6.2	5.2
	Heparin (%)	11.6	9.4
	Antiplatelet (%)	40.0	35.4
	Antihyperlipidemic (%)	9.3	5.2
	Insulin (%)	4.6	3.1
	Oral antidiabetics (%)	9.3	12.5
	Age (years)	93.2 ± 3.2	92.6 ± 3.4
**Risk factors**	Civil status (% of married person)	95.8	97.9
	Living with (number of person)	2.8 ± 1.2	2.6 ± 1.2
	Education (years)	4.2 ± 2.6	4.3 ± 2.6
	Tobacco exposure (%)	13.9	72.9
	Winsor index	1.0 ± 0.3	1.1 ± 0.3
	ABI code > 1 (%)	23.6	36.5
	Handrig index (kg)	14.3 ± 6.9	15.8 ± 7.
	Pase score (%)	38.6	52.1
	Sleep-alertness (%)	4.3	4.2
	BMI (kg/*m*^2^)	24.8 ± 4.7	25.1 ± 3.4
	MDS index	34.0 ± 3.9	34.5 ± 3.3

#### Ethical considerations

The Mugello study [31] was conducted in agreement with the principles of the Helsinki Declaration on Clinical Research involving human beings (1964) and was approved by the Don Carlo Gnocchi Foundation Ethics Committee. Informed written consent was obtained from all participants or from their delegates before their inclusion in the original study.

### Experimental methods

#### Sample collection

Blood samples were collected after overnight fasting, centrifuged at 2000*g* for 10 min at 4°C, and stored in aliquots at −80° until analyses, following standardized operating procedure as described in Bernini et al*.* [47].

#### NMR experiments

Serum samples were prepared for NMR analysis as described by Bernini et al. [47] and acquired using a Bruker 600 MHz spectrometer (Bruker BioSpin s.r.l., Germany). NOESY 1D presat (one-dimensional NOESY) experiments were used to measure selectively low and high molecular weight molecules. Metabolites, lipoproteins, lipid fractions and sub-fractions were assigned, identified, and quantified using the AVANCE IVDr (Clinical Screening and In Vitro Diagnostics (IVD) research with B.I. Methods, Bruker BioSpin) [48], and the principal metabolites and main lipid fractions considered in this study are listed as follow: alanine, creatine, glutamic acid, glutamine, glycine, histidine, isoleucine, leucine, lysine, phenylalanine, proline, threonine, tyrosine, valine, acetic acid, citric acid, lactic acid, acetoacetic acid, pyruvic acid, glucose, main parameter (MP) triglycerides, main parameter (MP) cholesterol, main parameter (MP) LDL, main parameter (MP) HDL, main parameter (MP) Apo A1, main parameter (MP) Apo A2, and main parameter (MP) Apo B100.

A complete list of all lipid fractions and sub-fractions is presented in Supplementary Table S1.

### Statistical methods

#### Data pre-processing

Only covariates with less than 25% missing data were considered; missing data were imputed using a Random Forest approach as implemented in R package missForest [49]; default parameters were used. All variables were log-transformed before analysis. One sample (“F_C_138”) was excluded after a check of the spectra due to low quality shimming and remove from all subsequent analyses: the actual number of samples used in the present investigation is *n*=355.

### Extraction of metabolic information related to clinical variables

For each blood metabolite and lipid fraction, we extracted the variation coming from a given clinical covariate, i.e., one of the *M*=101 covariates (see Table 1) recorded, using the method proposed by Bartzis et al. [50]*.* This approach extracts, from each metabolite/lipid, the part of concentration that is associated with a given covariate, thus implicitly adjusting for the remaining *M*-1 covariates. The rationale is that different metabolites/lipids sharing similar correlation/association with the same clinical covariable tend to be close to each other in the network, thus providing a better representation of the underlying biological phenomena [50].

Briefly, be Y^(*p*)^ the (*n* × 1) vector of concentrations of the *p-*th metabolite or lipid component (with *p* = 1,2, ..., *P*) measured on *n*=355 subjects and be **X** the (*n* × *M*) matrix containing the *M*=101 clinical parameters (covariates) recorded on the *n* subjects. Let be X_*m*_the *n* × 1 vector containing the values for *m*-th clinical variables and X^(−*m*)^ = {X_1_, X_2_, …, X_*M* − 1_} the remaining *M*−1 clinical variables. The information ${\hat{\mathrm{Y}}}^{(p)}$of a metabolite or lipid component *p* associated with a specific clinical covariate *m* was estimated by regressing Y^(*p*)^ on **X** and retaining only the main effects and first-order interactions of covariate X_*m*_:

1 $$ {\hat{\mathrm{Y}}}^{(p)}={\hat{\beta}}^{(p)}{\mathrm{X}}_m+{\sum}_{\delta \in \varDelta }{\hat{\eta}}_{\delta}^{(p)}{\mathrm{X}}_m\circ {\prod}_{j=1}^{M-1}{\mathrm{X}}_j^{\delta_j} $$

where ${\sum}_{\delta \in \varDelta }{\hat{\eta}}_{\delta}^{(p)}{\mathrm{X}}_m\circ {\prod}_{j=1}^{M-1}{\mathrm{X}}_j^{\delta_j}$ models all main effects and high-order interactions in terms of clinical variables.

For each clinical parameter *m* the procedure is repeated for all *P* metabolites and lipid component to obtain *M* = 101 *n* × *P* data sets Y_*m*_ = {Y_(1)_,Y_(2)_, …, Y_(*p*)_} containing the *part of the measured* metabolite and lipid concentrations associated with each one of the 101 clinical parameters.

### Network analysis

#### Network concepts

We briefly review here some network concepts. A network is a graphical representation of the relationships between objects, called nodes [51]. In a biological network, the nodes are molecular components, like genes, proteins, or, like in this study, metabolites and lipid components. The (existence of a) relationship between two nodes (molecular components) is represented by an edge connecting the two nodes. The type of association among the molecular features can be diverse in nature: in a protein-protein interaction network, edges represent the existence of physical interaction between proteins; in a metabolite-metabolite association network in which two metabolites are connected if their concentration levels are correlated.

Mathematically, a network can be represented as an adjacency (also called connectivity) matrix **A**: the rows and columns of the **A** represent the nodes whereas the entries *a*_*ij*_ represent edges. A network is said to be *unweighted* if the edges *a*_*ij*_ describing the association between node *i* and *j* are either 1 or 0:

2 $$ {a}_{ij}=\left\{\begin{array}{c}1\kern2.5em \mathrm{if}\kern1.25em \left(i,j\right)\ \mathrm{are}\ \mathrm{associated}\\ {}0\kern9.25em \mathrm{otherwise}\end{array}\right. $$

If the strength or magnitude of the relationship can be quantified, a weight can be given to the edge; then, the network is said to be *weighted*: in this case, the elements of a weighted adjacency matrix **A** are real numbers indicating the strength of the interaction, and can vary, for instance, in the [−1, 1] range if the correlation is used as an index for the association.

#### Reconstruction of metabolite and lipid association networks

The Probabilistic Context Likelihood of Relatedness (PCLRC) [52] algorithm was used to build metabolite and lipid association networks using Spearman correlation as a measure of association [53]. The algorithm allows robust estimation of correlation employing a resampling strategy in combination with a modified version of the Context Likelihood of Relatedness (CLR) [54] to remove non-significant background correlations. The algorithm returns a probability matrix **P** with values between 0 and 1 that was used to filter significant correlation *r*_*ij*_ between pairs of metabolites /lipids. In particular

3 $$ {r}_{ij}=\left\{\begin{array}{c}{r}_{ij}\kern0.75em \mathrm{if}\kern1em {p}_{ij}\ge 0.90\\ {}0\kern0.99em \mathrm{if}\kern0.6em {p}_{ij}<0.90\end{array}\ \right. $$

We built a metabolite/lipids association network for each of the 101 *n*_*1*_ × *P* (for women) and *n*_*2*_ × *P* (for men) data sets Y_*m*_={Y_(1)_,Y_(2)_, ..., Y_(*p*)_} containing the *part of the measured* metabolite and lipid concentrations associated with each one of the 101 clinical parameters. We analyzed data for males and females separately, obtaining a total of 202 metabolite/lipids association networks that were divided into 5 categories (*S*= diseases, bio-humoral parameters, familiarity diseases, risk factors, and drug treatments).

#### *Network differential connectivity analysis*

Each node in a network can be characterized using measures that can be derived from the patterns of its association. A very common measure is the node degree or connectivity [55, 56], that is the number of its connection. For a *p*×*p* network **A**, the connectivity of the node *i* is given by:

4 $$ {\chi}_i=\sum \limits_{j>i}\mid {a}_{ij}\mid $$

Given a network A, the connectivity ${\chi}_i^A$for metabolite/lipid i is defined as

5 $$ {\chi}_i^A=\left({\sum}_{j=1}^J\left|{r}_{ij}\right|\right)-1 $$

If the network is unweighted, it holds 0<χ_i_<p−1. If the network is weighted, the range of the connectivity depends on the nature of the association measure. If the absolute value of the correlation is used, like in this study, χ_i_ still ranges between 0 and p−1, in which case, it means that the molecular feature represented by node ai is perfectly correlated with all other nodes in the network.

Two networks **A** and **B** associated with two different conditions or groups (such as those built from men and women samples, or from samples from case-control patients) can be compared, implementing a so-called differential network analysis [28, 57].

The differential connectivity (${\Delta }_i^{A,B}$) of a metabolite/lipid *i* between two networks A and B is defined as

6 $$ {\Delta }_i^{A,B}={\chi}_i^A-{\chi}_i^B $$

**Fig. 2:**
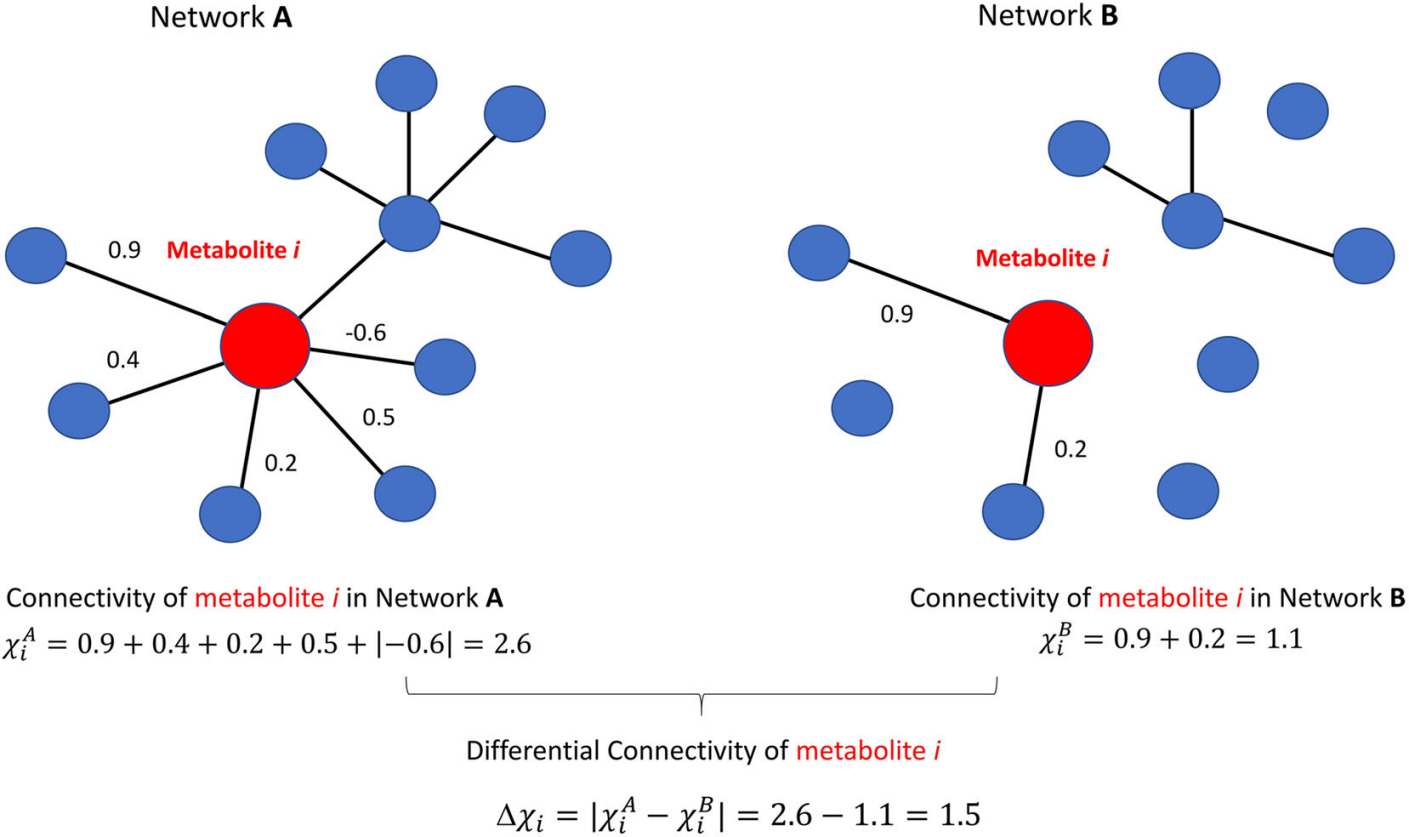
Graphical illustration of the concept of node connectivity and differential connectivity used in this study. Each node represents a molecular feature (metabolite, lipid). The edge connecting two nodes represent the esistence of an association between two nodes, in this case expressed by correlation; the weight of the edge is given by the (absolute value) of the correlation. Figure adapted from [53]

The concept of differential connectivity is exemplified in Fig. 2.

#### Estimation of the statistical significance of differential network connectivity

The statistical significance of the differential connectivity (${\Delta }_{i,k}^{A,B}$), was assessed by means of a permutation-test. Briefly, the columns of every Y_*m*_ matrix are independently permuted to obtain a permutated matrix **X**_(*k*)_whose column mean and variance are unchanged but the association between the elements of different columns is destroyed.

For each metabolite/lipid the differential connectivity was calculated for networks *a* and *b* built from the permuted data:

7 $$ {\Delta }_{i,k}^{A,B}={\chi}_{i,k}^A-{\chi}_{i,k}^B $$

and the overall procedure was repeated *k*=100 times to create a null distribution *D*_*i*_ of permutated differential connectivity values. The significance of a given differential connectivity value ${\Delta }_i^{A,B}$ (calculated on the original data) was calculated as a *P*-value using the following formula (#() indicates the number of elements):

8 $$ P- value=\frac{1+\#\left(\left|{D}_i\right|>\left|{\Delta }_i^{A,B}\right|\right)}{k} $$

#### Multivariate analysis of association networks

Covariance simultaneous component analysis (COVSCA) [58] was performed to analyze simultaneously the (dis)similarities of the sets of K=101 metabolite and lipid association networks. The K association matrices are modeled as the number of low-dimensional prototypes (*L* << K):

9 $$ {\mathbf{S}}_k\cong {\sum}_{l=1}^L{c}_{kl}{\mathbf{Z}}_l{\mathbf{Z}}_l^T $$

where *c*_*kl*_≥ 0 (l = 1, 2, …, *L*) are weight coefficients and${\mathbf{Z}}_l{\mathbf{Z}}_l^T$are the prototypical covariance matrices that characterize the loadings set **Z** of dimension *J* × *R*_*l*_ that hold together for all *C*_*k*_.

The COVSCA model was fitted separately for both women's and men's data with 3 rank-1 prototype matrices (*R*=3) as the best compromise between the goodness-of-fit (82.7%) and model complexity.

In COVSCA, each network becomes a point in an *R* dimensional space and thus the method provides a methodology to represent and visualize a large number of networks in a way akin to standard principal component analysis: points (i.e., metabolite-lipid association networks) close in the R-dimensional space share similar characteristics, i.e., similar patterns of correlation among lipids and metabolites. The relative importance of each metabolite/lipid in shaping the observed network differences is given by the loadings that can also be interpreted in a PCA fashion.

#### Clustering

T-distributed stochastic neighbor embedding (t-SNE) [59] was applied on the 3-dimensional COVSCA scores to visualize detectable similarities and clusters among  the networks.

#### Software

Calculations were performed using MATLAB (version 2018b R 9.5.0.9) an R (version 3.3.2). The R code for the PCLRC algorithm and the code to perform differential connectivity analysis are available at the link: www.semantics.systemsbiology.nlunder the SOFTWARE tab.

## Results and discussion

### Sex-specific differences of metabolite-lipid association networks

Metabolites and lipidic components take part in many metabolic processes; association networks, that quantify and visualize the interrelationships between molecular features, are representations of the complex web of biochemical reactions and pathways underlying the functioning of an organism: changes in the network structure can be considered to mirror alterations or re-modulation of the underlying network of metabolic reactions [28]. The sex-specific serum metabolite-lipid component association networks were built using separately samples from nonagenarian women (*n*_*1*_=259) and men (*n*_*2*_=96), to avoid confounding due to sex. Previous results obtained on this study cohort have observed sex-associated differences between men and women characteristics [60, 61]. However, although the interplay between sex differences and age-related differences has not been explored fully, accumulating evidence of sex dimorphism in the disease susceptibility [62, 63] aging and longevity phenotypes [64, 65] suggests the necessity of analyzing separately men and women data.

**Fig. 3:**
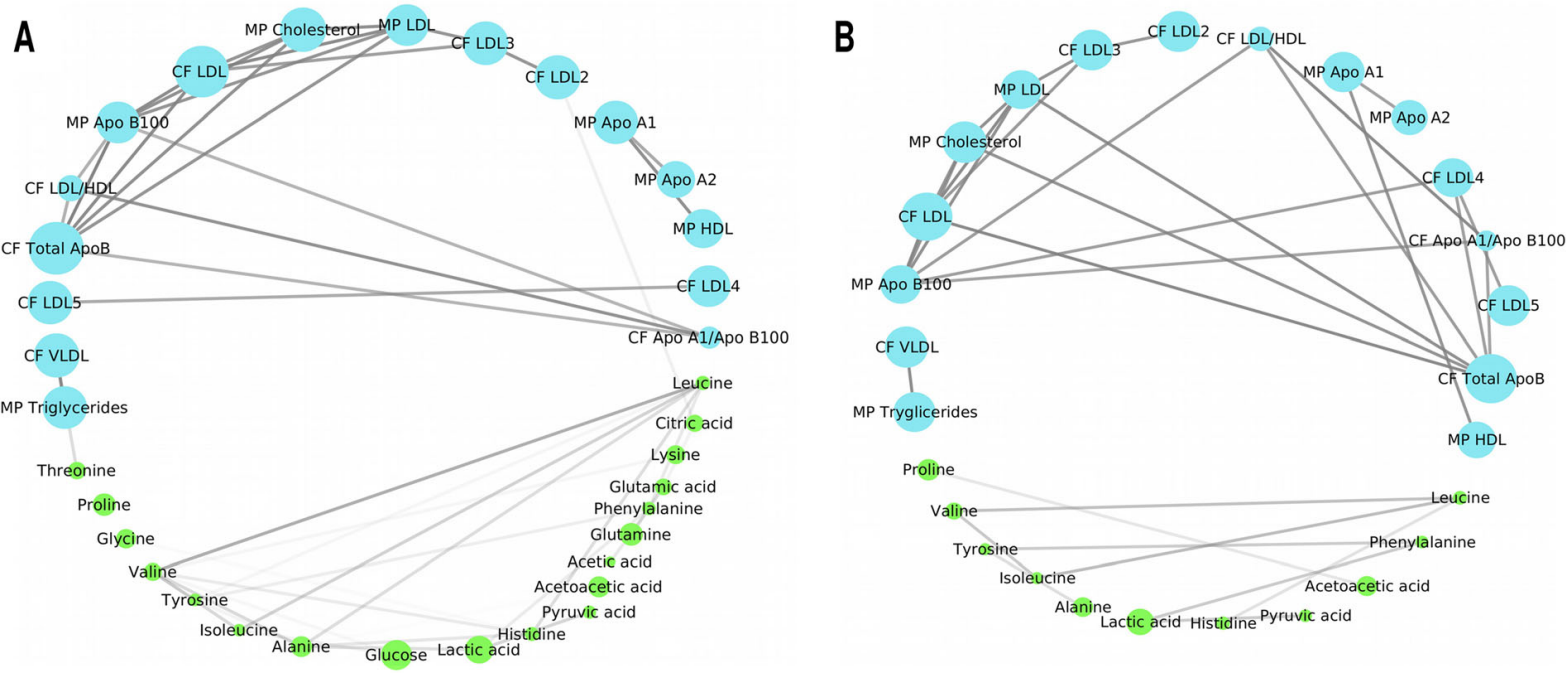
Metabolite-lipid association networks for women (A) and men (B). Nodes are colored according to compounds’ classification groups, light blue for lipid main parameters (MP) and calculated figures (CF), and light green for metabolites. Edges represent correlation with |r| ≥ 0.6 and their width depends on the likelihood of the connections (see Eq. (3)). For sake of simplicity only metabolites, lipid main parameters, and calculated figures are shown

The women- and men-specific networks have markedly different topology. The women-specific network (Fig. 3A) is more densely connected, for what concern metabolite-metabolite associations, with respect to the men-specific one (Fig. 3B). In both networks, lipidic components (lipoproteins, lipid fractions, and sub-fractions) have a strongly inter-connected structure although different lipid species are involved. We quantified and assessed the statistical differences of the metabolite-lipid association networks specific to men and women using node connectivity which quantifies the number and the strength of metabolite-lipid associations, thus representing the importance of given metabolite/lipid in the network.

**Fig. 4:**
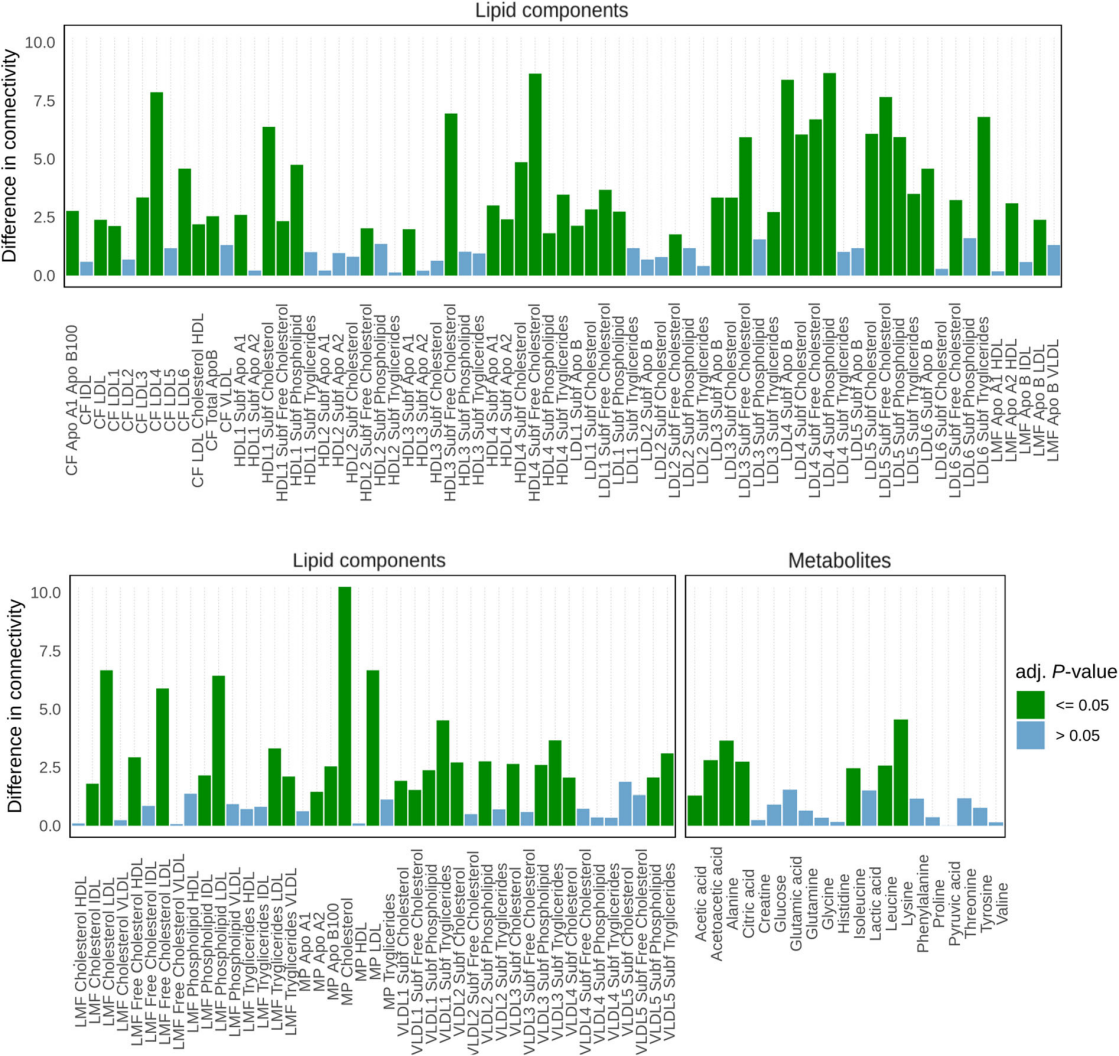
Differential connectivity (Eq. (6); see Fig. 2 for an overview) from the differential network analysis of sex-related networks (men vs women) given in Fig. 3. For each metabolite and lipid component, adjusted *P*-values (Benjamini-Hochberg) are given.

Differential network analysis results are given in Fig. 4. Seven out of 20 metabolites and 67 out of 114 lipoproteins and lipid fractions and sub-fractions have different connectivity patterns between men and women networks (adjusted *P*-value ≤ 0.05). Among metabolites, only alanine, isoleucine, leucine, lysine, citric acid, acetoacetic acid, acetic acid, showed altered connectivity, indicating re-modulation of amino acids and ketone bodies’ metabolism, a result consistent with other studies focusing on the network-based analysis of the sex-specific difference in metabolite profiles in men and women [66–68]. In elderly women, re-modulation of amino acid metabolism results in a decreased level of branched amino acids (BCAAs) with respect to men [69, 70], and this phenomenon is associated with larger muscle mass loss, depending not only on a reduction in physical activity but also on a reduction of hormone activity, on an inadequate diet, and on the presence chronic diseases [69, 70]. Consistently with these observations, we found disruption of the association between alanine and leucine (present in women but not in the men network): leucine stimulates muscle protein synthesis [70–72] but is an important nitrogen donor for alanine biosynthesis [73].

Disruption of acetoacetic acid and acetic acid connectivity patterns suggests a re-modulation of ketone bodies’ metabolism: acetic acid levels associate with prolonged fasting and diabetic ketosis, potentially frequent in elderly people with metabolic diseases [74], and, in women, with post-menopausal downregulation of gonadotropin which induces glycolytic dysregulation, resulting in a shift from physiological aerobic metabolism to a ketogenic phenotype [75, 76]*.*

### Similarities and dissimilarities in different sex-related clinical variables-specific networks

We explored in a comprehensive way the relationships between metabolite-lipid association networks and the different 101 clinical covariates describing, for each subject, either the presence of a specific pathophysiological condition, familiarity diseases, pharmacological treatments, risk factors or the levels of 51 bio-humoral parameters (such as complete blood count, mean cell volume, hemoglobin, thyroid hormones. A complete list is reported in Table 1). For each of the 20 metabolites and 114 lipid fractions and sub-fractions, we first extracted the part of the observed variation of the concentration associated with a given clinical covariate (see Eq. (1)), and then we built association networks using only this fraction of the concentration, obtaining 101 (women) + 101 (men) different metabolite-lipid association networks. The rationale is that metabolites/lipids sharing similar relationships with a given covariate tend to be close to each other in the network, and this can provide a clearer representation of the underlying biological phenomena.

**Fig. 5:**
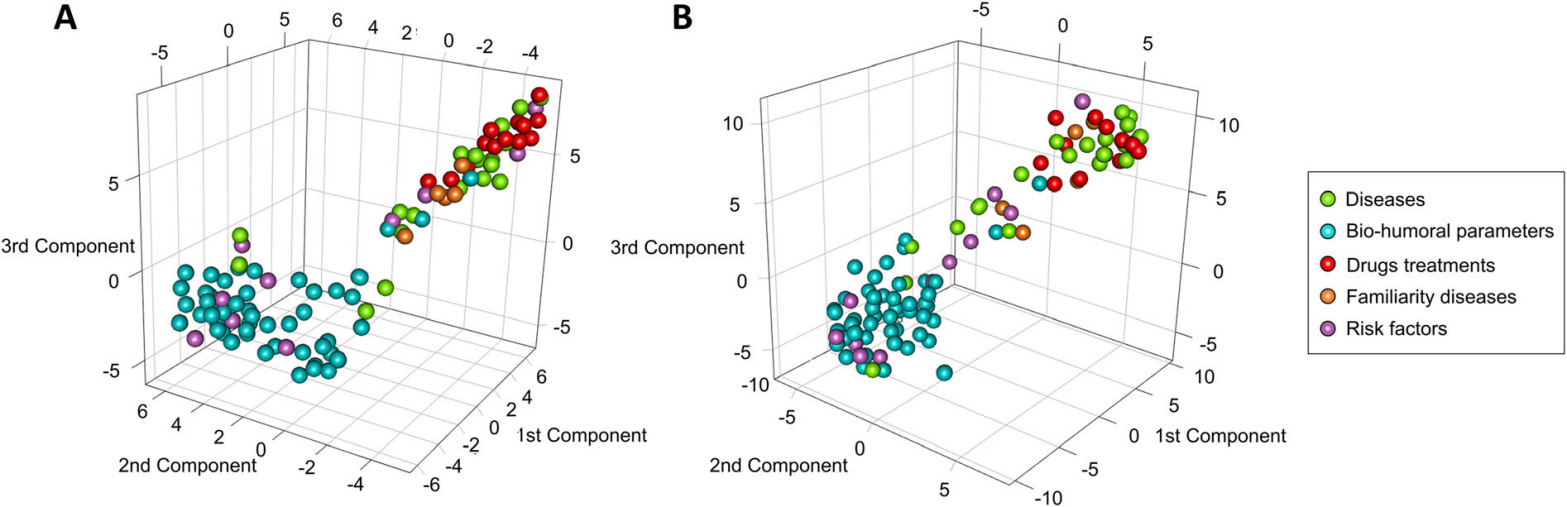
Multivariate analysis of the metabolite-lipid association networks associated with the 101 clinical covariates (see Table 1) for women (A) and (B). The 101 + 101 networks are analyzed using Covariance Simultaneous Component Analysis (see the “Multivariate analysis of association networks” section). Each sphere corresponds to a network and is coloured according to the clinical variable-specific set: green colour corresponding to diseases, blue colour to bio-humoral parameters, red colour to drugs treatments, light brown colour to familiarity diseases and light violet colour to risk factors. Clustering is performed on the COVSCA score using t-SNE. Metabolite and lipid importance are given in Fig. 6

Since is not practically possible to compare all 202 networks individually, we used a multivariate component method (COVSCA, see Methods) to model and visualize the (dis)similarity and the relationships among the networks in combination with clustering; results are shown for women in Fig. 5A and for men in Fig. 5B, where each dot represents a metabolite-lipid association network specific to a given covariate.

For both women and men, networks specific to bio-humoral parameters separate from the networks specific to disease-related networks, indicating that the patterns of association among metabolites and lipid fractions and sub-fractions with these covariates are markedly different from those associated with comorbidity. Given a (clinical/biochemical) covariate, the statistical procedure employed can be seen as a correction procedure for confounders: in this light, the bio-humoral associated networks can be seen as representing metabolite/lipid relationship in a healthy condition. Networks associated with diseases tend to cluster with the networks related to ongoing pharmacological treatment of the same diseases suggesting the existence of oh shared information among these two groups of networks. Networks associated with risk factors (like age, smoking habits, and BMI) are scattered, indicating great heterogeneity and possibly reflecting that many risk scores are composite indexes summarizing both clinical and molecular features.

**Fig. 6:**
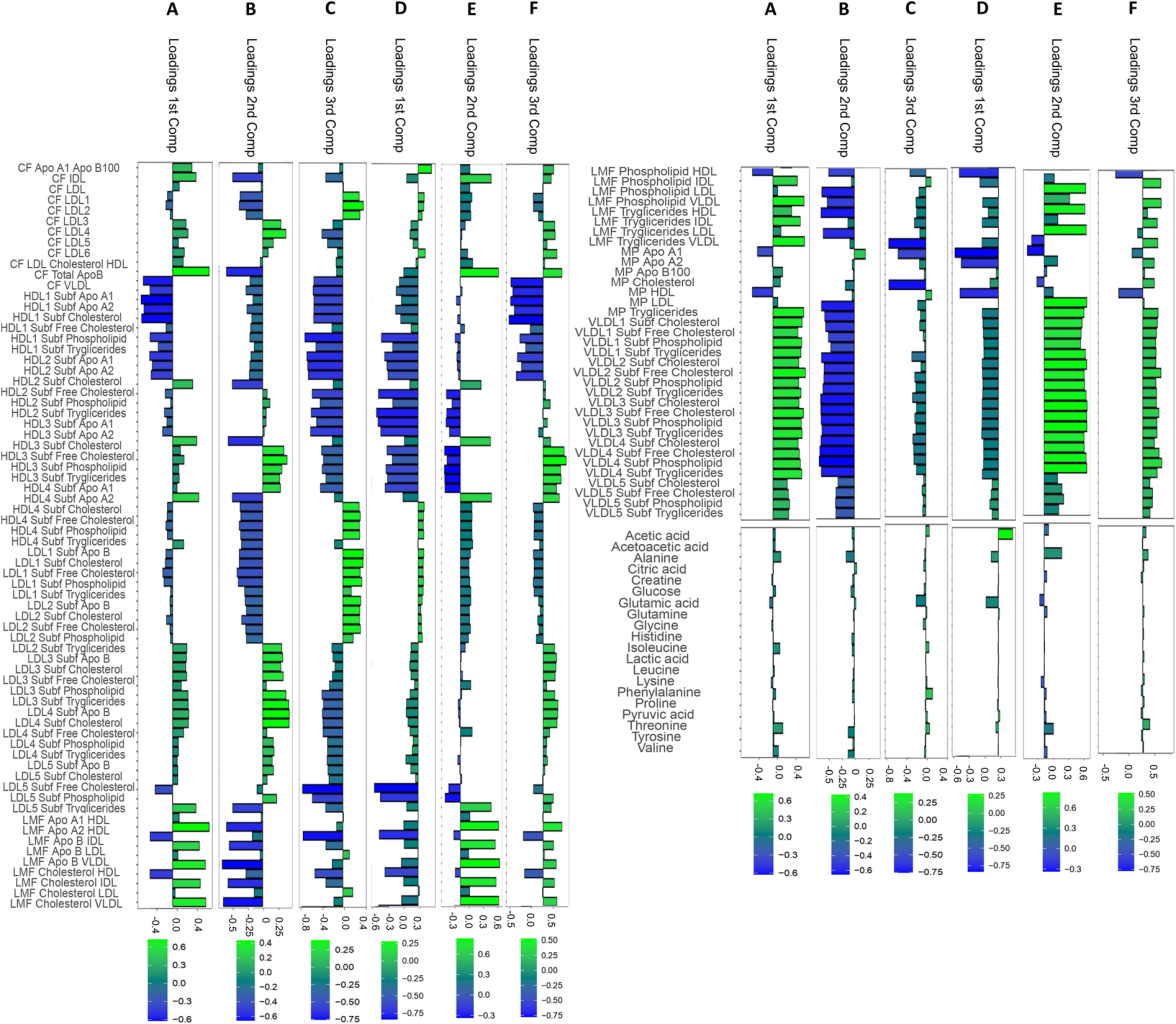
Multivariate analysis of the metabolite-lipid association networks associated with the 101 clinical covariates (see Table 1) for women (A) and (B). The loadings give the importance of the metabolite and lipids to explain the patterns of network (dis)similarity observed in Fig. 6. Panel A–C: loadings for the analysis of women networks (Fig. 5A); Panel D–F: loadings for the analysis of men networks (Fig. 5B)

The relative importance of metabolites and lipidic components to explain the network clustering shown in Fig. 5A and B are given in Fig. 6A, B, and C for women and in Fig. 6D, E, F for men and can be interpreted as in standard principal component analysis (PCA). For both men and women, the first two components of the COVSCA model, explaining the variability within bio-humoral associated networks, are dominated LDL and VLDL lipid sub-fractions, while the third component, explaining the variability within disease-associated networks, is dominated by LDL and HDL. Our results show that different patterns of association between LDL and HDL fractions or interaction thereof are associated with comorbidity in this study cohort: LDL and HDL are not only associated with cardiovascular disease [77, 78], type II diabetes [79, 80], peripheral vascular disease [81, 82] and hypertension [83, 84] but also with dementia [85, 86] and cancer [87, 88]: the strong lipidic signature, suggest a potential different manifestation and response to health diseases in the elderly population [89, 90].

Overall, metabolites do not seem to play a significant role in shaping the observed difference among networks: only acetic acid, alanine, glutamine, and, less strongly, pyruvic acid have a relevant contribution to the model. Glucogenic amino acids (glutamine and alanine) have been associated with the regulation of aging and aging-related diseases [69]: in particular, altered levels of glutamine have been associated with higher intima-media thickness of carotid artery and, consequently, with coronary artery atherosclerosis, causing cardio-vascular syndromes [91, 92], and could be responsible for the increase of the activity of the osteoclasts resulting in a reduction of the bone mineral density [93]. Alanine and acetic acids are correlated with protein-energy malnutrition in aged people [94], metabolic syndromes [95], and, in post-menopausal women, are correlated with *a cellular ketogenic phenotypic change* [75, 76].

Differential clinical networks analysis in nonagenarian women and men

**Fig. 7:**
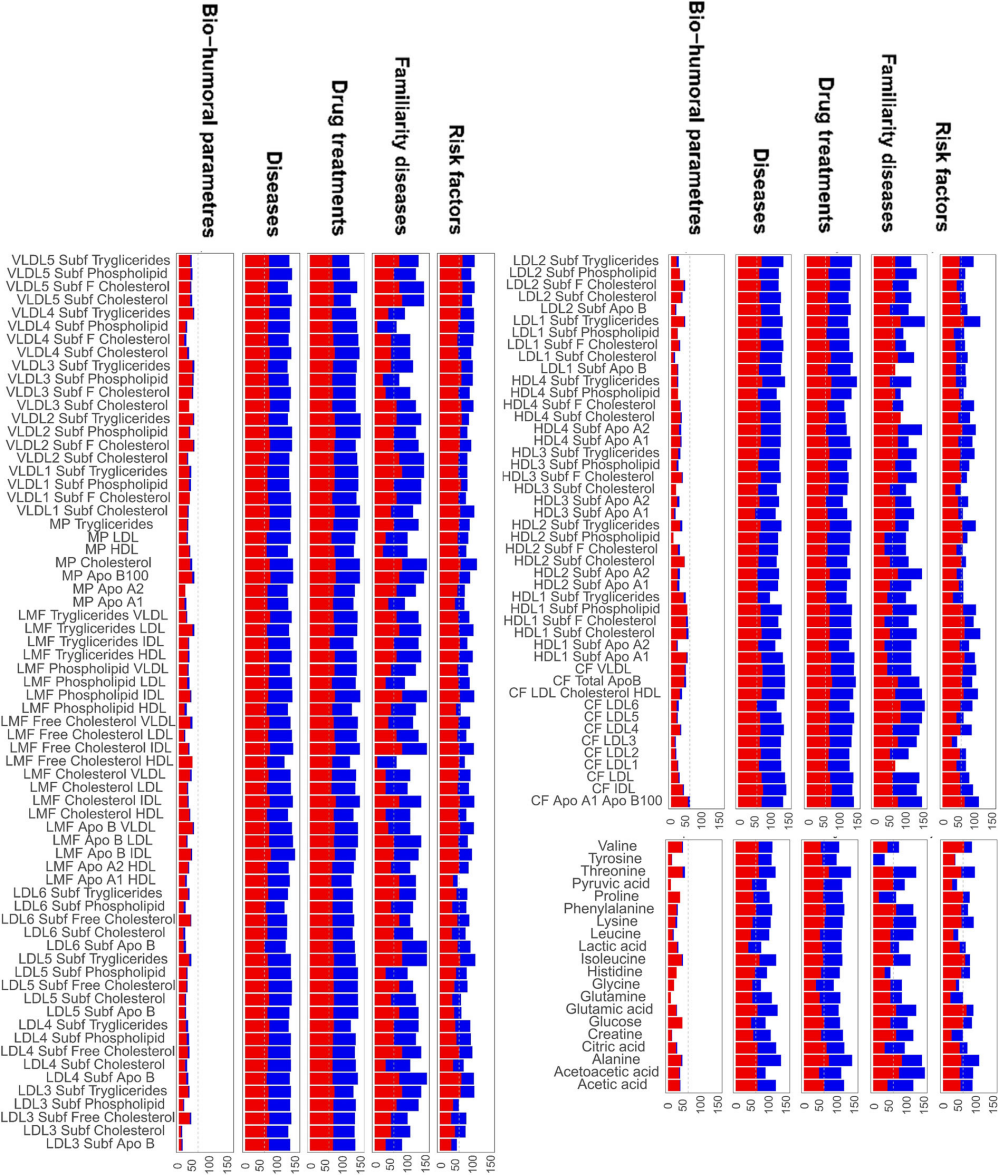
Results of pairwise comparison of the 101 + 101 networks associated with clinical the covariates. The percentage of time that a metabolite or lipid is found to be significantly differentially connected (adjusted *P*-value < 0.05) between any two networks belonging to the clinical covariate of the same type is shown. The (overlapping) red bars correspond to the women-related clinical variable-specific networks and blue bars correspond to the men-related clinical variable-specific networks

Starting from the observation that the metabolite/lipids association networks associated with the same type of covariate (bio-humoral parameters, diseases, drugs treatments, risk factors, and familiarity diseases) tend to share similar but not identical correlation patterns (see Fig. 7), we performed a pairwise comparison among the networks related to similar covariates. For each comparison, we recorded the significantly differentially connected metabolites (*P*-value adjusted ≤ 0.05) and lipoproteins/lipid fractions and sub-fractions (see Eq. (8)) for each comparison. We retained for further investigation only those molecular features that were found to be significant in more than 70% of the comparisons. Results are shown in Fig. 7. For both men and women bio-humoral parameter networks, we observe that very few metabolites and lipid features show different connectivity among different networks, indicating the similarity of these networks.

The comparison of disease**-**associated networks shows that for both men and women the full spectrum of (measured) lipids is associated with the differences among networks, suggesting their central role in pathophysiological mechanisms and in their resolutions; lipids are not only engaged in inter- and intra-cellular signaling regulation pathways but are also able to orchestrate inflammation processes and to restore the homeostasis [96], which may explain also the strong lipid signature observed also in drug-treatment associated networks. In women risk factor-networks lipid components, in particular, VLDL, plays a major role: in post-menopausal women, VLDL is negatively associated with estrogens [97–99], and this is related to increased risk of cardiovascular diseases [100, 101], myocardial infarction [102], and hyperlipidemia [103]: its association with diverse risk factors is worthy of mention since VLDL is actually being reconsidered as a potential biomarker [78, 104, 105].

The role of metabolites in explaining network differences is more nuanced, and this probably reflects the large biochemical diversity of these molecules [106]. In diseases-specific networks, key differentiating metabolites are ketone bodies, BCAAs, threonine, and tyrosine (in women networks only), and alanine: this can reflect the association with particular diseases, such as type 1 and 2 diabetes mellitus [91, 107–110], sarcopenia [69, 71, 72, 111] and cognitive impairment [112, 113]. Glutamine, glucose, proline, and BCAAs, increasing their metabolic activity, could be biomarkers to predict the emergence of neurodegenerative diseases [114], type 2 diabetes mellitus, and obesity conditions [91, 108, 109], and sarcopenia [69, 71, 72].

**Fig. 8:**
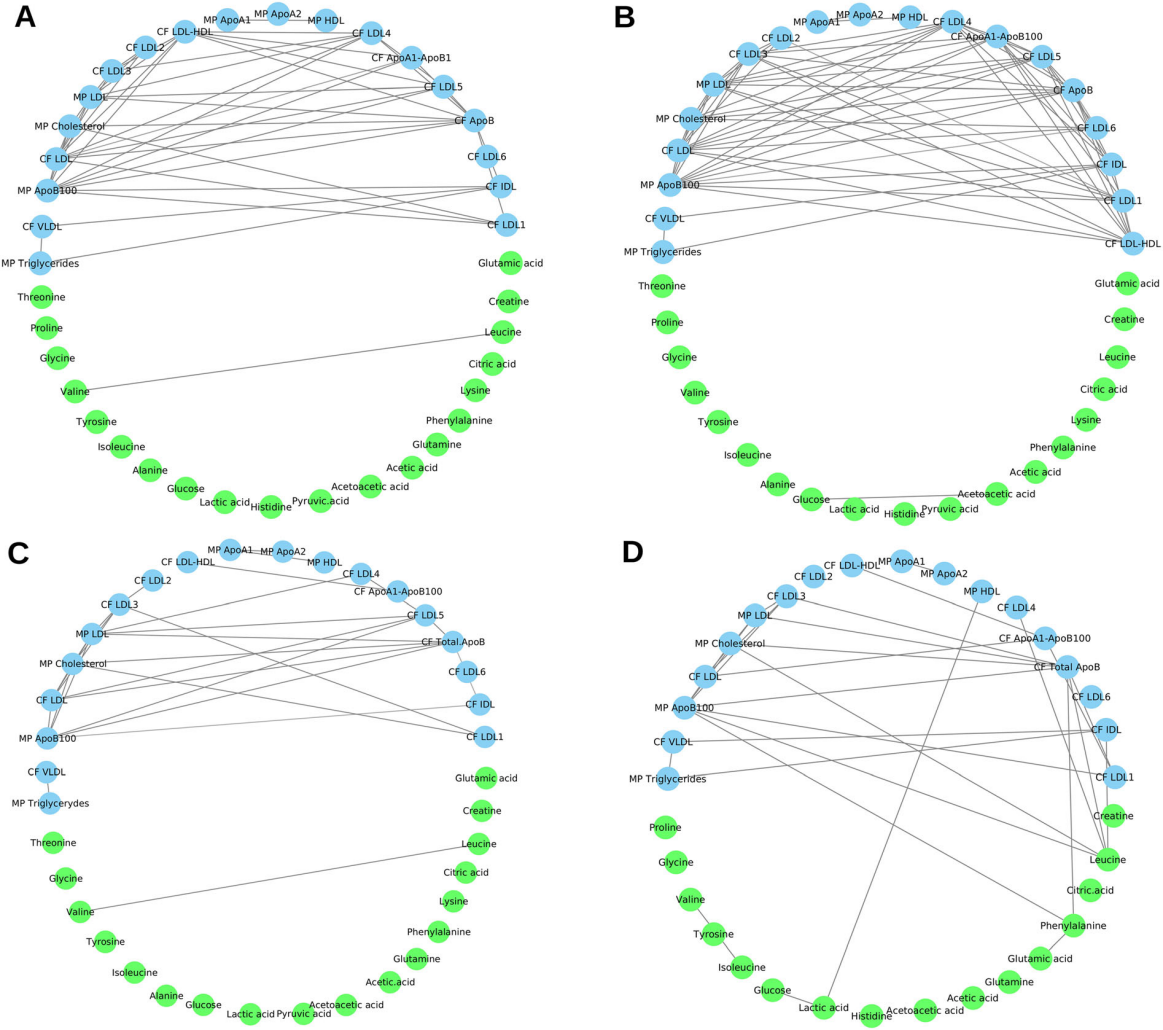
Metabolite-lipid networks associated with peripheral vascular disease (A: women, B: men) and Diabetes (C: women, D: men). Nodes are colored according to compounds’ classification groups, light blue color for lipid main parameters (MP) and calculated figures (CF), and light green color for metabolites. Edges represent correlation with |r| ≥ 0.6 and their width depends on the likelihood of the connections. For sake of simplicity only metabolites, lipid main parameters (MP), and calculated figures (CF) are shown

As an example, we show the networks associated with peripheral vascular disease and diabetes in both women and men (Fig. 8) which are two of the most common comorbidities among the subjects in this study (see Table 1).

When comparing the peripheral vascular disease association networks of women (Fig. 8A) and men (Fig. 8B), differential connectivity of leucine with lipidic components and among lipids component: while the latter has a strong association with peripheral vascular disease [82, 115], the remodulation of association between leucine and lipids suggests the existence an interplay between amino acids and lipids.

The difference in lipid correlation observed in diabetes-specific women (Fig. 8C) and men networks (Fig. 8D) further points to sex-specific differences in lipid metabolism [116, 117]. In particular, in women we observe a disruption of the correlation between acetic acids and glucose; it is known that acetic acid can lower glucose level, and can improve insulin resistance and metabolic abnormalities in the atherogenic prediabetic state [118]: the mechanism are not yet fully understood [119] and, as shown here, may be differentially regulated in men and women.

## Conclusions

In this study, we presented a differential network analysis approach to highlight sex-related metabolic differences in cohort of nonagenarian subjects. Comparing the networks of nonagenarian women and men, we observed that lipids, branched chains amino acids, alanine, and ketone bodies show significant differences in connectivity in the two groups. In particular, we observed that lipids not only play a central role in the structural robustness of the network but also are directly associated with the intrinsic dynamic metabolic sex-related changes. The same approach was also applied to identify, in the disease**-**associated networks built in nonagenarian women and men, significantly differentially connected metabolites and lipoproteins/lipid fractions and sub-fractions. Our results show the importance of the lipid components in diseases, drug treatments, and familiarity disease, indicating their ability to participate in many pathophysiological mechanisms in nonagenarians; in the women's disease-specific networks, the rewiring of metabolic activity involves ketone bodies, branched chains amino acids, threonine, and tyrosine. In conclusion, this study provides information about the structure of sex-related networks in nonagenarians, contributing to elucidate the impact of gender on human physiology and pathophysiology in the elderly population and showcases how network analysis may provide a valuable tool in gender medicine.

## Supplementary information

ESM 1 (DOCX 18.7 kb)

## Data Availability

On request

## Code availability

Code available upon request to E.S.
