# Supplementary material for: Lipid and metabolite correlation networks specific to clinical and biochemical covariate show differences associated with sexual dimorphism in a cohort of nonagenarians
Source: GeroScience. 2021 Jul 29;44(2):1109–28. doi: 10.1007/s11357-021-00404-3 (PMC9135919; doi:10.1007/s11357-021-00404-3)
Supplement: Supplementary file 1 — (DOCX 18.7 kb) [file 11357_2021_404_MOESM1_ESM.docx]

**Supplementary Materials:**

**Table S1: Complete list of lipid fractions and sub-fractions considered**

| **Lipid fractions and sub-fractions** |
| --- |
| Calcutated Figures (CF) LDL/HDL |
| Calcutated Figures (CF) Apo A1/Apo B100 |
| Calcutated Figures (CF) Total ApoB |
| Calcutated Figures (CF) VLDL |
| Calcutated Figures (CF) IDL |
| Calcutated Figures (CF) LDL |
| Calcutated Figures (CF) LDL1 |
| Calcutated Figures (CF) LDL2 |
| Calcutated Figures (CF) LDL3 |
| Calcutated Figures (CF) LDL4 |
| Calcutated Figures (CF) LDL5 |
| Calcutated Figures (CF) LDL6 |
| Lipoprotein Main Fractions (LMF) Triglycerides VLDL |
| Lipoprotein Main Fractions (LMF) Triglycerides IDL |
| Lipoprotein Main Fractions (LMF) Triglycerides LDL |
| Lipoprotein Main Fractions (LMF) Triglycerides HDL |
| Lipoprotein Main Fractions (LMF) Cholesterol VLDL |
| Lipoprotein Main Fractions (LMF) Cholesterol IDL |
| Lipoprotein Main Fractions (LMF) Cholesterol LDL |
| Lipoprotein Main Fractions (LMF) Cholesterol HDL |
| Lipoprotein Main Fractions (LMF) Free Cholesterol VLDL |
| Lipoprotein Main Fractions (LMF) Free Cholesterol IDL |
| Lipoprotein Main Fractions (LMF) Free Cholesterol LDL |
| Lipoprotein Main Fractions (LMF) Free Cholesterol HDL |
| Lipoprotein Main Fractions (LMF) Phospholipids VLDL |
| Lipoprotein Main Fractions (LMF) Phospholipids IDL |
| Lipoprotein Main Fractions (LMF) Phospholipids LDL |
| Lipoprotein Main Fractions (LMF) Phospholipids HDL |
| Lipoprotein Main Fractions (LMF) Apo A1 HDL |
| Lipoprotein Main Fractions (LMF) Apo A2 HDL |
| Lipoprotein Main Fractions (LMF) Apo B VLDL |
| Lipoprotein Main Fractions (LMF) Apo B IDL |
| Lipoprotein Main Fractions (LMF) Apo B LDL |
| VLDL1 Subfractions Triglycerides |
| VLDL2 Subfractions Triglycerides |
| VLDL3 Subfractions Triglycerides |
| VLDL4 Subfractions Triglycerides |
| VLDL5 Subfractions Triglycerides |
| VLDL1 Subfractions Cholesterol |
| VLDL2 Subfractions Cholesterol |
| VLDL3 Subfractions Cholesterol |
| VLDL4 Subfractions Cholesterol |
| VLDL5 Subfractions Cholesterol |
| VLDL1 Subfractions Free Cholesterol |
| VLDL2 Subfractions Free Cholesterol |
| VLDL3 Subfractions Free Cholesterol |
| VLDL4 Subfractions Free Cholesterol |
| VLDL5 Subfractions Free Cholesterol |
| VLDL1 Subfractions Phospholipids |
| VLDL2 Subfractions Phospholipids |
| VLDL3 Subfractions Phospholipids |
| VLDL4 Subfractions Phospholipids |
| VLDL5 Subfractions Phospholipids |
| LDL1 Subfractions Triglycerides |
| LDL2 Subfractions Triglycerides |
| LDL3 Subfractions Triglycerides |
| LDL4 Subfractions Triglycerides |
| LDL5 Subfractions Triglycerides |
| LDL6 Subfractions Triglycerides |
| LDL1 Subfractions Cholesterol |
| LDL2 Subfractions Cholesterol |
| LDL3 Subfractions Cholesterol |
| LDL4 Subfractions Cholesterol |
| LDL5 Subfractions Cholesterol |
| LDL6 Subfractions Cholesterol |
| LDL1 Subfractions Free Cholesterol |
| LDL2 Subfractions Free Cholesterol |
| LDL3 Subfractions Free Cholesterol |
| LDL4 Subfractions Free Cholesterol |
| LDL5 Subfractions Free Cholesterol |
| LDL6 Subfractions Free Cholesterol |
| LDL1 Subfractions Phospholipids |
| LDL2 Subfractions Phospholipids |
| LDL3 Subfractions Phospholipids |
| LDL4 Subfractions Phospholipids |
| LDL5 Subfractions Phospholipids |
| LDL6 Subfractions Phospholipids |
| LDL1 Subfractions Apo B |
| LDL2 Subfractions Apo B |
| LDL3 Subfractions Apo B |
| LDL4 Subfractions Apo B |
| LDL5 Subfractions Apo B |
| LDL6 Subfractions Apo B |
| HDL1 Subfractions Triglycerides |
| HDL2 Subfractions Triglycerides |
| HDL3 Subfractions Triglycerides |
| HDL4 Subfractions Triglycerides |
| HDL1 Subfractions Cholesterol |
| HDL2 Subfractions Cholesterol |
| HDL3 Subfractions Cholesterol |
| HDL4 Subfractions Cholesterol |
| HDL1 Subfractions Free Cholesterol |
| HDL2 Subfractions Free Cholesterol |
| HDL3 Subfractions Free Cholesterol |
| HDL4 Subfractions Free Cholesterol |
| HDL1 Subfractions Phospholipids |
| HDL2 Subfractions Phospholipids |
| HDL3 Subfractions Phospholipids |
| HDL4 Subfractions Phospholipids |
| HDL1 Subfractions Apo A1 |
| HDL2 Subfractions Apo A1 |
| HDL3 Subfractions Apo A1 |
| HDL4 Subfractions Apo A1 |
| HDL1 Subfractions Apo A2 |
| HDL2 Subfractions Apo A2 |
| HDL3 Subfractions Apo A2 |
| HDL4 Subfractions Apo A2 |
